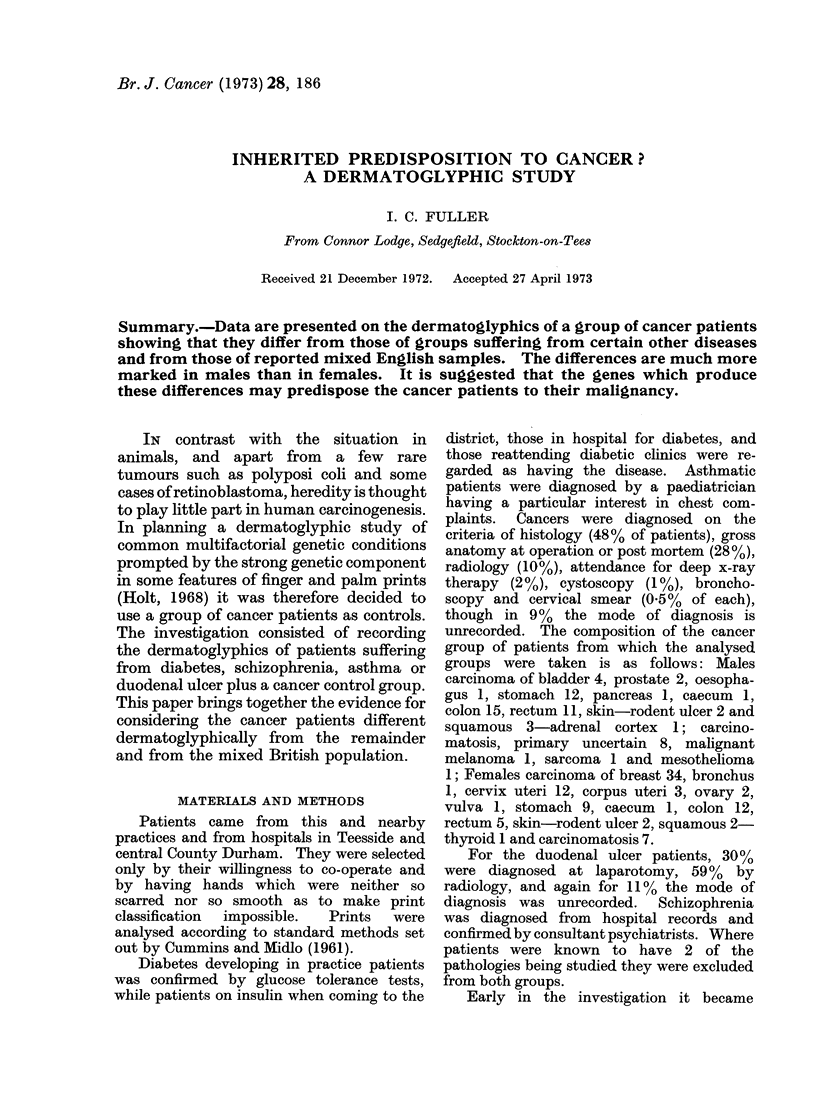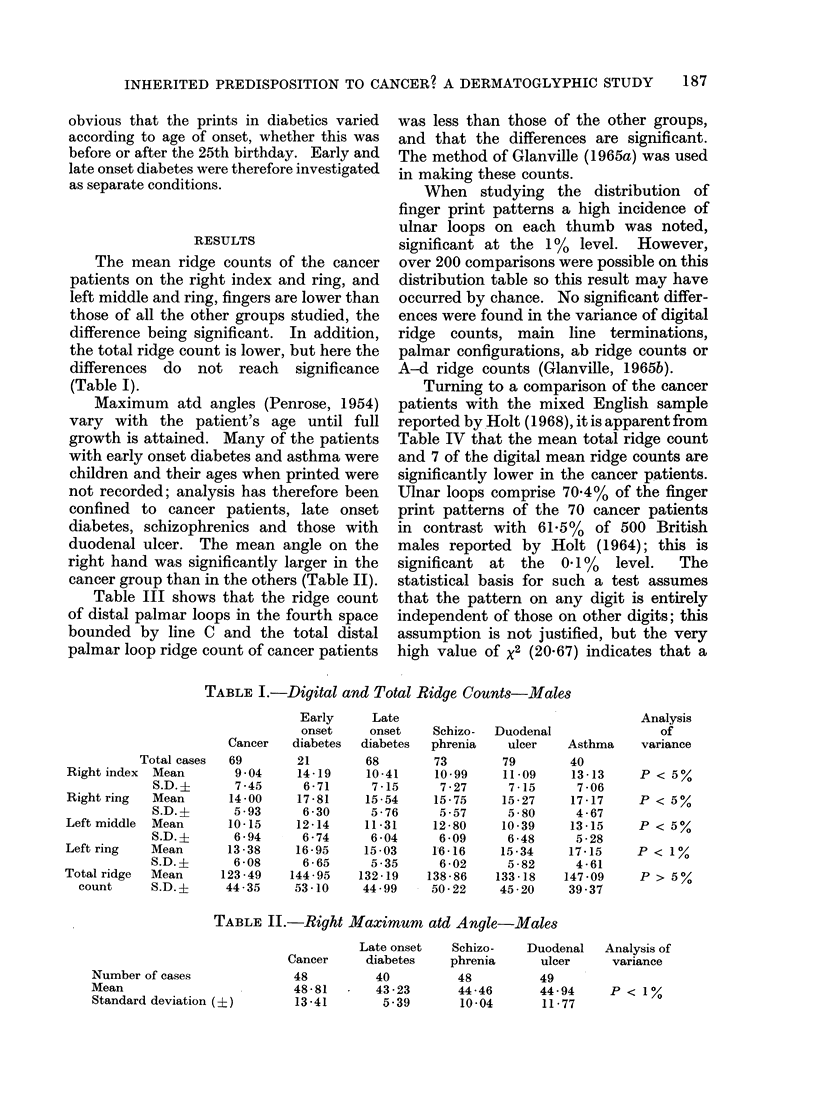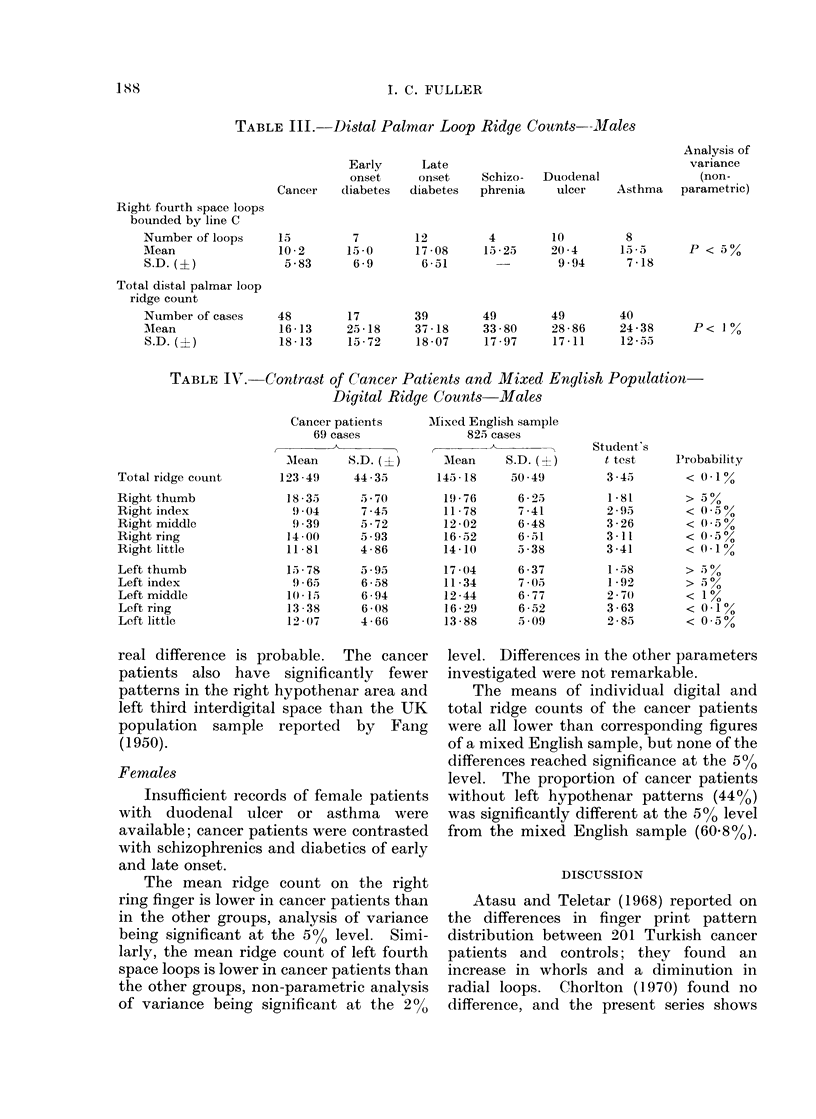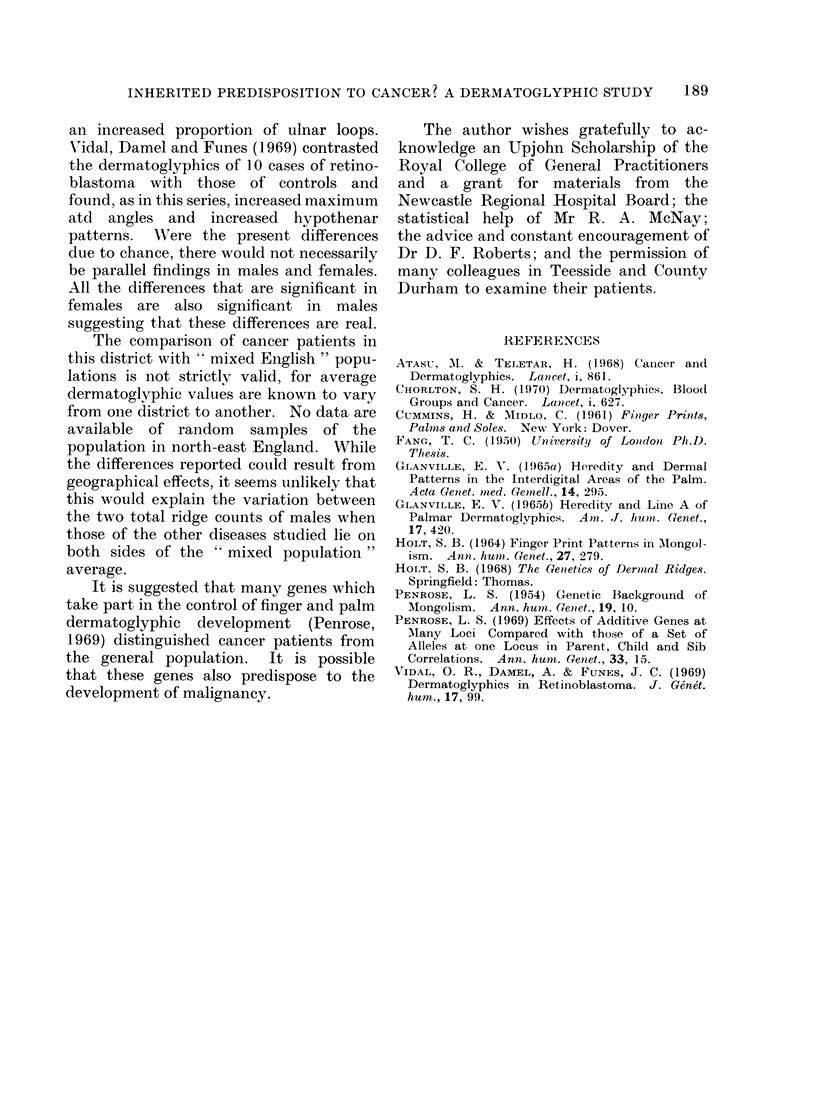# Inherited Predisposition to Cancer? A Dermatoglyphic Study

**DOI:** 10.1038/bjc.1973.135

**Published:** 1973-08

**Authors:** I. C. Fuller

## Abstract

Data are presented on the dermatoglyphics of a group of cancer patients showing that they differ from those of groups suffering from certain other diseases and from those of reported mixed English samples. The differences are much more marked in males than in females. It is suggested that the genes which produce these differences may predispose the cancer patients to their malignancy.


*Br. J. Cancer* (1973) **28**, 186

### INHERITED PREDISPOSITION TO CANCER? A DERMATOGLYPHIC STUDY

I. C. FULLER

*From Connor Lodge, Sedgefield, Stockton-on-Tees*

Received 21 December 1972. Accepted 27 April 1973

**Summary.—Data are presented on the dermatoglyphics of a group of cancer patients showing that they differ from those of groups suffering from certain other diseases and from those of reported mixed English samples. The differences are much more marked in males than in females. It is suggested that the genes which produce these differences may predispose the cancer patients to their malignancy.**

In contrast with the situation in animals, and apart from a few rare tumours such as polyposi coli and some cases of retinoblastoma, heredity is thought to play little part in human carcinogenesis. In planning a dermatoglyphic study of common multifactorial genetic conditions prompted by the strong genetic component in some features of finger and palm prints (Holt, 1968) it was therefore decided to use a group of cancer patients as controls. The investigation consisted of recording the dermatoglyphics of patients suffering from diabetes, schizophrenia, asthma or duodenal ulcer plus a cancer control group. This paper brings together the evidence for considering the cancer patients different dermatoglyphically from the remainder and from the mixed British population.

### MATERIALS AND METHODS

Patients came from this and nearby practices and from hospitals in Teesside and central County Durham. They were selected only by their willingness to co-operate and by having hands which were neither so scarred nor so smooth as to make print classification impossible. Prints were analysed according to standard methods set out by Cummins and Midlo (1961).

Diabetes developing in practice patients was confirmed by glucose tolerance tests, while patients on insulin when coming to the district, those in hospital for diabetes, and those reattending diabetic clinics were regarded as having the disease. Asthmatic patients were diagnosed by a paediatrician having a particular interest in chest complaints. Cancers were diagnosed on the criteria of histology (48% of patients), gross anatomy at operation or post mortem (28%), radiology (10%), attendance for deep x-ray therapy (2%), cystoscopy (1%), bronchoscopy and cervical smear (0·5% of each), though in 9% the mode of diagnosis is unrecorded. The composition of the cancer group of patients from which the analysed groups were taken is as follows: Males carcinoma of bladder 4, prostate 2, oesophagus 1, stomach 12, pancreas 1, caecum 1, colon 15, rectum 11, skin—rodent ulcer 2 and squamous 3—adrenal cortex 1; carcinomatosis, primary uncertain 8, malignant melanoma 1, sarcoma 1 and mesothelioma 1; Females carcinoma of breast 34, bronchus 1, cervix uteri 12, corpus uteri 3, ovary 2, vulva 1, stomach 9, caecum 1, colon 12, rectum 5, skin—rodent ulcer 2, squamous 2—thyroid 1 and carcinomatosis 7.

For the duodenal ulcer patients, 30% were diagnosed at laparotomy, 59% by radiology, and again for 11% the mode of diagnosis was unrecorded. Schizophrenia was diagnosed from hospital records and confirmed by consultant psychiatrists. Where patients were known to have 2 of the pathologies being studied they were excluded from both groups.

Early in the investigation it became obvious that the prints in diabetics varied according to age of onset, whether this was before or after the 25th birthday. Early and late onset diabetes were therefore investigated as separate conditions.

### RESULTS

The mean ridge counts of the cancer patients on the right index and ring, and left middle and ring, fingers are lower than those of all the other groups studied, the difference being significant. In addition, the total ridge count is lower, but here the differences do not reach significance (Table I).

Maximum atd angles (Penrose, 1954) vary with the patient's age until full growth is attained. Many of the patients with early onset diabetes and asthma were children and their ages when printed were not recorded; analysis has therefore been confined to cancer patients, late onset diabetes, schizophrenics and those with duodenal ulcer. The mean angle on the right hand was significantly larger in the cancer group than in the others (Table II).

Table III shows that the ridge count of distal palmar loops in the fourth space bounded by line C and the total distal palmar loop ridge count of cancer patients was less than those of the other groups, and that the differences are significant. The method of Glanville (1965*a*) was used in making these counts.

When studying the distribution of finger print patterns a high incidence of ulnar loops on each thumb was noted, significant at the 1% level. However, over 200 comparisons were possible on this distribution table so this result may have occurred by chance. No significant differences were found in the variance of digital ridge counts, main line terminations, palmar configurations, ab ridge counts or A–d ridge counts (Glanville, 1965*b*).

Turning to a comparison of the cancer patients with the mixed English sample reported by Holt (1968), it is apparent from Table IV that the mean total ridge count and 7 of the digital mean ridge counts are significantly lower in the cancer patients. Ulnar loops comprise 70·4% of the finger print patterns of the 70 cancer patients in contrast with 61·5% of 500 British males reported by Holt (1964); this is significant at the 0·1% level. The statistical basis for such a test assumes that the pattern on any digit is entirely independent of those on other digits; this assumption is not justified, but the very high value of $\chi^2$ (20·67) indicates that a

TABLE I.—*Digital and Total Ridge Counts—Males*

| | | Cancer | Early onset diabetes | Late onset diabetes | Schizo-phrenia | Duodenal ulcer | Asthma | Analysis of variance |
|---|---|---|---|---|---|---|---|---|
| | Total cases | 69 | 21 | 68 | 73 | 79 | 40 | |
| Right index | Mean | 9·04 | 14·19 | 10·41 | 10·99 | 11·09 | 13·13 | $P < 5\%$ |
| | S.D.± | 7·45 | 6·71 | 7·15 | 7·27 | 7·15 | 7·06 | |
| Right ring | Mean | 14·00 | 17·81 | 15·54 | 15·75 | 15·27 | 17·17 | $P < 5\%$ |
| | S.D.± | 5·93 | 6·30 | 5·76 | 5·57 | 5·80 | 4·67 | |
| Left middle | Mean | 10·15 | 12·14 | 11·31 | 12·80 | 10·39 | 13·15 | $P < 5\%$ |
| | S.D.± | 6·94 | 6·74 | 6·04 | 6·09 | 6·48 | 5·28 | |
| Left ring | Mean | 13·38 | 16·95 | 15·03 | 16·16 | 15·34 | 17·15 | $P < 1\%$ |
| | S.D.± | 6·08 | 6·65 | 5·35 | 6·02 | 5·82 | 4·61 | |
| Total ridge | Mean | 123·49 | 144·95 | 132·19 | 138·86 | 133·18 | 147·09 | $P > 5\%$ |
| count | S.D.± | 44·35 | 53·10 | 44·99 | 50·22 | 45·20 | 39·37 | |

TABLE II.—*Right Maximum atd Angle—Males*

| | Cancer | Late onset diabetes | Schizo-phrenia | Duodenal ulcer | Analysis of variance |
|---|---|---|---|---|---|
| Number of cases | 48 | 40 | 48 | 49 | |
| Mean | 48·81 | 43·23 | 44·46 | 44·94 | $P < 1\%$ |
| Standard deviation (±) | 13·41 | 5·39 | 10·04 | 11·77 | |

TABLE III.—*Distal Palmar Loop Ridge Counts—Males*

| | Cancer | Early onset diabetes | Late onset diabetes | Schizophrenia | Duodenal ulcer | Asthma | Analysis of variance (non-parametric) |
|---|---|---|---|---|---|---|---|
| Right fourth space loops bounded by line C | | | | | | | |
| Number of loops | 15 | 7 | 12 | 4 | 10 | 8 | |
| Mean | 10·2 | 15·0 | 17·08 | 15·25 | 20·4 | 15·5 | $P < 5\%$ |
| S.D. (±) | 5·83 | 6·9 | 6·51 | — | 9·94 | 7·18 | |
| Total distal palmar loop ridge count | | | | | | | |
| Number of cases | 48 | 17 | 39 | 49 | 49 | 40 | |
| Mean | 16·13 | 25·18 | 37·18 | 33·80 | 28·86 | 24·38 | $P < 1\%$ |
| S.D. (±) | 18·13 | 15·72 | 18·07 | 17·97 | 17·11 | 12·55 | |

TABLE IV.—*Contrast of Cancer Patients and Mixed English Population—Digital Ridge Counts—Males*

| | Cancer patients 69 cases | | Mixed English sample 825 cases | | | |
|---|---|---|---|---|---|---|
| | Mean | S.D. (±) | Mean | S.D. (±) | Student's $t$ test | Probability |
| Total ridge count | 123·49 | 44·35 | 145·18 | 50·49 | 3·45 | < 0·1% |
| Right thumb | 18·35 | 5·70 | 19·76 | 6·25 | 1·81 | > 5% |
| Right index | 9·04 | 7·45 | 11·78 | 7·41 | 2·95 | < 0·5% |
| Right middle | 9·39 | 5·72 | 12·02 | 6·48 | 3·26 | < 0·5% |
| Right ring | 14·00 | 5·93 | 16·52 | 6·51 | 3·11 | < 0·5% |
| Right little | 11·81 | 4·86 | 14·10 | 5·38 | 3·41 | < 0·1% |
| Left thumb | 15·78 | 5·95 | 17·04 | 6·37 | 1·58 | > 5% |
| Left index | 9·65 | 6·58 | 11·34 | 7·05 | 1·92 | > 5% |
| Left middle | 10·15 | 6·94 | 12·44 | 6·77 | 2·70 | < 1% |
| Left ring | 13·38 | 6·08 | 16·29 | 6·52 | 3·63 | < 0·1% |
| Left little | 12·07 | 4·66 | 13·88 | 5·09 | 2·85 | < 0·5% |

real difference is probable. The cancer patients also have significantly fewer patterns in the right hypothenar area and left third interdigital space than the UK population sample reported by Fang (1950).

*Females*

Insufficient records of female patients with duodenal ulcer or asthma were available; cancer patients were contrasted with schizophrenics and diabetics of early and late onset.

The mean ridge count on the right ring finger is lower in cancer patients than in the other groups, analysis of variance being significant at the 5% level. Similarly, the mean ridge count of left fourth space loops is lower in cancer patients than the other groups, non-parametric analysis of variance being significant at the 2% level. Differences in the other parameters investigated were not remarkable.

The means of individual digital and total ridge counts of the cancer patients were all lower than corresponding figures of a mixed English sample, but none of the differences reached significance at the 5% level. The proportion of cancer patients without left hypothenar patterns (44%) was significantly different at the 5% level from the mixed English sample (60·8%).

### DISCUSSION

Atasu and Teletar (1968) reported on the differences in finger print pattern distribution between 201 Turkish cancer patients and controls; they found an increase in whorls and a diminution in radial loops. Chorlton (1970) found no difference, and the present series shows an increased proportion of ulnar loops. Vidal, Damel and Funes (1969) contrasted the dermatoglyphics of 10 cases of retinoblastoma with those of controls and found, as in this series, increased maximum atd angles and increased hypothenar patterns. Were the present differences due to chance, there would not necessarily be parallel findings in males and females. All the differences that are significant in females are also significant in males suggesting that these differences are real.

The comparison of cancer patients in this district with " mixed English " populations is not strictly valid, for average dermatoglyphic values are known to vary from one district to another. No data are available of random samples of the population in north-east England. While the differences reported could result from geographical effects, it seems unlikely that this would explain the variation between the two total ridge counts of males when those of the other diseases studied lie on both sides of the " mixed population " average.

It is suggested that many genes which take part in the control of finger and palm dermatoglyphic development (Penrose, 1969) distinguished cancer patients from the general population. It is possible that these genes also predispose to the development of malignancy.

The author wishes gratefully to acknowledge an Upjohn Scholarship of the Royal College of General Practitioners and a grant for materials from the Newcastle Regional Hospital Board; the statistical help of Mr R. A. McNay; the advice and constant encouragement of Dr D. F. Roberts; and the permission of many colleagues in Teesside and County Durham to examine their patients.

### REFERENCES

Atasu, M. & Teletar, H. (1968) Cancer and Dermatoglyphics. *Lancet*, i, 861.

Chorlton, S. H. (1970) Dermatoglyphics, Blood Groups and Cancer. *Lancet*, i, 627.

Cummins, H. & Midlo, C. (1961) *Finger Prints, Palms and Soles*. New York: Dover.

Fang, T. C. (1950) *University of London Ph.D. Thesis*.

Glanville, E. V. (1965*a*) Heredity and Dermal Patterns in the Interdigital Areas of the Palm. *Acta Genet. med. Gemell.*, **14**, 295.

Glanville, E. V. (1965*b*) Heredity and Line A of Palmar Dermatoglyphics. *Am. J. hum. Genet.*, **17**, 420.

Holt, S. B. (1964) Finger Print Patterns in Mongolism. *Ann. hum. Genet.*, **27**, 279.

Holt, S. B. (1968) *The Genetics of Dermal Ridges*. Springfield: Thomas.

Penrose, L. S. (1954) Genetic Background of Mongolism. *Ann. hum. Genet.*, **19**, 10.

Penrose, L. S. (1969) Effects of Additive Genes at Many Loci Compared with those of a Set of Alleles at one Locus in Parent, Child and Sib Correlations. *Ann. hum. Genet.*, **33**, 15.

Vidal, O. R., Damel, A. & Funes, J. C. (1969) Dermatoglyphics in Retinoblastoma. *J. Génét. hum.*, **17**, 99.